# Unravelling the components of diffuse scattering using deep learning

**DOI:** 10.1107/S2052252523009521

**Published:** 2024-01-01

**Authors:** Chloe A. Fuller, Lucas S. P. Rudden

**Affiliations:** a Swiss-Norwegian Beamlines, ESRF, Grenoble, France; b Institute of Bioengineering, EPFL, Lausanne, Switzerland; Lund University, Sweden; Keele University, United Kingdom

**Keywords:** diffuse scattering, deep learning, short-range order, Pix2Pix generative adversarial networks, molecular form factors, computational modelling, molecular crystals, disorder

## Abstract

Many technologically important material properties are underpinned by disorder and short-range structural correlations; therefore, elucidating structure–property relationships in functional materials requires understanding both the average and the local structures. The latter information is contained within diffuse scattering but is challenging to exploit, particularly in single-crystal systems. Separation of the diffuse scattering into its constituent components can greatly simplify analysis and allows for quantitative parameters describing the disorder to be extracted directly. Here, a deep-learning method, DSFU-Net, is presented based on the Pix2Pix generative adversarial network, which takes a plane of diffuse scattering as input and factorizes it into the contributions from the molecular form factor and the chemical short-range order. DSFU-Net was trained on 198 421 samples of simulated diffuse scattering data and performed extremely well on the unseen simulated validation dataset in this work. On a real experimental example, DSFU-Net successfully reproduced the two components with a quality sufficient to distinguish between similar structural models based on the form factor and to refine short-range-order parameters, achieving values comparable to other established methods. This new approach could streamline the analysis of diffuse scattering as it requires minimal prior knowledge of the system, allows access to both components in seconds and is able to compensate for small regions with missing data. DSFU-Net is freely available for use and represents a first step towards an automated workflow for the analysis of single-crystal diffuse scattering.

## Abstract 2

A deep-learning method is applied to separate the components of diffuse scattering from chemical short-range order and the molecular form factor. The method is validated against a large simulated dataset and further tested on a real example, resulting in output components of sufficient quality to use for quantitative analysis.

## 1. Introduction

The properties of functional materials often depend on the presence of defects and their arrangement at a local scale. Measurement of the diffuse scattering arising from such disorder provides a means to probe the local structure, facilitating understanding, and ultimately control, of the distribution of defects in order to tune the useful properties (Simonov *et al.*, 2020 ▸). While there are fairly routine practices for studying diffuse scattering in powder samples, *e.g.* pair distribution function (PDF) analysis (Billinge, 2019 ▸), the study of single-crystal diffuse scattering has remained much more niche, despite having a larger information content.

Typical modelling strategies for single-crystal diffuse scattering fall into four categories: analytical, 3D-PDF, direct Monte Carlo (MC) and reverse MC simulations. The latter two are most commonly used because they are the most generalisable; however, the modelling process is very challenging, being highly sensitive to the inputs, either of the disorder model or the parameterization of the algorithm. The development of the 3D-ΔPDF method (Weber & Simonov, 2012 ▸) and the *Yell* program (Simonov *et al.*, 2014*b* ▸) allows for the direct refinement of local correlations in real space, but the consequent necessity of using a Fourier transform requires careful measurement and pre-treatment of the scattering data.

**Figure 1:**
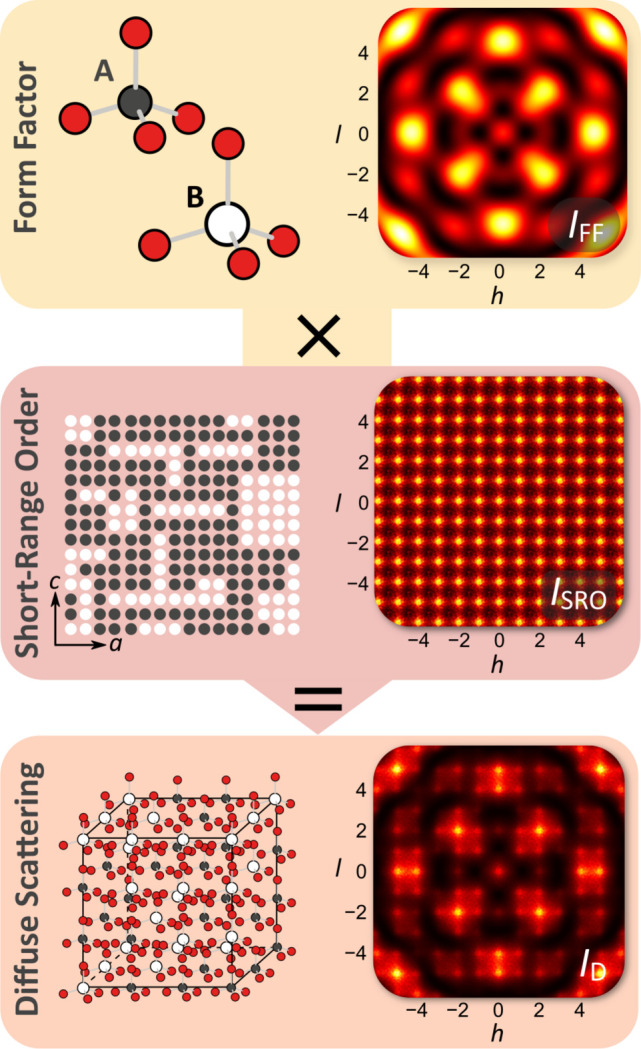
The diffuse scattering components of binary substitutional disorder with one disordered site per unit cell. The site can be occupied by either molecule A or molecule B, as shown in the top panel. Molecule A has a smaller central atom, the red atoms sit closer to the central atom and the scattering power is lower than that of molecule B. The difference between their molecular form factors causes structured scattering shown by *I*_FF_. The middle panel illustrates SRO, *i.e.* whether molecule A is more likely to be found next to molecule B or *vice versa*. It gives rise to a periodic grid of maxima, *I*_SRO_. The bottom panel shows the whole disordered crystal structure and the total diffuse scattering, *I*_D_.

While analytical approaches are limited in their applicability, they can provide a complete description of disorder for certain systems. Analytical models allow the diffuse scattering to be split into its constituent components (Krivoglaz, 1996 ▸), meaning that each one can be analysed separately, greatly simplifying the problem. In the case of pure binary substitutional disorder, with one disordered site per unit cell, the diffuse scattering can be simplified such that it is a product of just two components: one arising from the absolute squared difference in molecular form factors, *I*_FF_, and one from the chemical short-range order, *I*_SRO_, correlations between sites. Fig. 1 ▸ illustrates this factorization.

Schmidt & Neder (2017 ▸) showed that, in such systems, *I*_SRO_ can be obtained by dividing the diffuse scattering by the known form factor difference squared, or, in the case where this is not known, by dividing by an average form factor squared. The resulting function can be projected into one reciprocal-space unit cell, and the Warren–Cowley SRO parameters (Warren *et al.*, 1951 ▸) can be extracted directly from it through a least-squares refinement, providing a quantitative description of local correlations. With the same analytical basis, Chodkiewicz *et al.* (2016 ▸) was able to use *I*_FF_ to refine the relative orientations of the disordered molecules of an organic salt, leading to an improved average structure model.

The diffuse scattering cannot be factorized into these two components numerically. While the SRO component can be obtained from the data (Schmidt & Neder, 2017 ▸), access to the form factor requires the SRO to be solved by other methods first. An alternative approach would be to exploit the different characteristics of each component: *I*_FF_ is a slowly varying continuous function with a symmetry related to the point group of the disordered portion of the structure, while *I*_SRO_ is a periodic pattern that is often discontinuous, containing sharp Bragg-like features.

The last few years have witnessed a revolution in the application of machine-learning techniques to previously unsolved problems. Deep learning, in particular, has been used to make huge strides in a plethora of fields: for example, in biophysics with solving protein structure from sequence (Jumper *et al.*, 2021 ▸), in large language models offering AI chatbot assistants capable of nuanced conversation on any topic (OpenAI, 2023 ▸) and in computer vision where complex images can be generated given text prompts (Rombach *et al.*, 2022 ▸).

In crystallography, convolutional neural networks (CNNs) have been used to extract lattice parameters and space groups from X-ray diffraction patterns (Chakraborty & Sharma, 2022 ▸; Aguiar *et al.*, 2019 ▸), perform phase identification from powder diffraction (Lee *et al.*, 2020 ▸), classify diffraction images based on scattering features (Wang *et al.*, 2017 ▸), detect Bragg spots (Ke *et al.*, 2018 ▸; Hao *et al.*, 2023 ▸; Liu *et al.*, 2022 ▸), fill in gaps in data collected on area detectors (Chavez *et al.*, 2022 ▸), and solve simple protein structures from single-crystal diffraction (Pan *et al.*, 2023 ▸). Variational autoencoders have been applied to elucidate the phase composition of thin films from scattering data (Banko *et al.*, 2021 ▸); find structure–property relationships, *e.g.* band gaps, from powder diffraction data (Lee *et al.*, 2022 ▸); and even predict new structures with specific band gaps (Ren *et al.*, 2022 ▸). These are all examples of how neural networks can learn to extract a wide range of crucial underlying features of a dataset given appropriate training. In this context, deep-learning methods are well suited to solving the issue of decomposing diffuse scattering data into *I*_SRO_ and *I*_FF_. Since our ultimate goal is to translate input images (reconstructed planes of diffuse scattering) into topologically related images, we opted to apply a Pix2Pix generative adversarial network (Pix2PixGAN) (Isola *et al.*, 2018 ▸), which has demonstrated its strength in a variety of image-processing tasks, such as in converting satellite imagery to digital road maps.

This article presents the curation of a large simulated training dataset; the design, training and validation of a tailored Pix2PixGAN; and its subsequent successful application to a real experimental example.

## 2. Methods

### 2.1. Creating a dataset

Our Pix2PixGAN required both input scattering data and the corresponding ground truth (GT) *I*_SRO_ or *I*_FF_ output for training. A typical problem such as this requires tens of thousands of samples for adequate training and to ensure that the network will be generalisable to unseen problems. However, the amount of available data on real systems where similar analysis has been performed is extremely small (Schmidt & Neder, 2017 ▸; Chodkiewicz *et al.*, 2016 ▸). It was, therefore, necessary to use a simulated dataset to train the networks. A mathematical description of the two components is given below.

For a crystal with one disordered site per unit cell, occupied by either molecule A or B (a molecule could also be an atom or molecular fragment), the *I*_FF_(**Q**) component of the diffuse scattering comes from the difference in molecular form factors between A and B: 

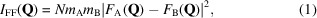

where *N* is the number of unit cells, and *m*_A_ and *m*_B_ are the average concentrations of A and B. *F*_A_(**Q**) is the molecular form factor of molecule A, equal to 

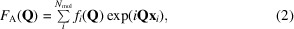

where *N*_mol_ is the number of atoms in the molecule; **x**_*i*_ and *f*_*i*_(**Q**) are the atomic position, in Cartesian coordinates, and atomic form factor of atom *i*, respectively; and **Q** is the scattering vector in units of Å^−1^. The chemical SRO component of the diffuse scattering is given by 

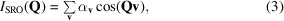

where **v** is the intermolecular vector between two sites in the crystal and the sum is over all possible vectors. The term α_**v**_ is the Warren–Cowley SRO parameter (Warren *et al.*, 1951 ▸) for **v**, defined as 

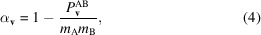

with 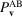 equal to the pair probability of finding a molecule of type B at a vector **v** from the molecule A. The overall diffuse scattering is given by the product: 

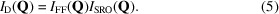

To obtain a sufficiently general dataset that encompasses all the factors that affect both components of the diffuse scattering, we simulated the scattering of many different pairs of molecules [*F*_A_(**Q**), *F*_B_(**Q**)] distributed on a range of lattices (**v**) with varying concentrations (*m*_A_, *m*_B_) and pair probabilities ( 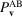 ).

To collate a list of A and B species, we extracted all examples from an online molecular-fragment library (Guzei, 2014 ▸), excluding those that were too similar, amounting to a diverse set of 58 molecules. A further four single atoms were added to the list as well as eight variations on the perovskite unit cell to include at least some extended structure types in the dataset. Forty-three of the molecules were randomly rotated about the Cartesian axes to expand the dataset further. Molecules were grouped according to their size and shape; details for this whole procedure are provided in Section 1.1 of the supporting information. A small selection of molecules in each group was set aside to be included in a validation dataset of roughly 5–10% the size of the training dataset to test the network’s generalizability. Within each group, a list of molecule pairs was made using all possible combinations, resulting in a training dataset of 1049 pairs of molecules and a validation set of 98 pairs.

For the SRO component, lists of the Warren–Cowley SRO parameters for each intermolecular vector (up to a certain cutoff, see Section 1.3 of the supporting information), consistent with the concentrations of A and B, were generated. Since the SRO parameters, α_**v**_ in equation (3), are highly interdependent, it is not possible to just use random values to give a realistic configuration. Instead, we specified three target parameters, α_[001]_, α_[010]_ and α_[100]_, along each crystallographic axis, and generated an atomic configuration consistent with these values using an MC simulation (further information can be found in Section 1.2 of the supporting information). The remaining SRO parameters were then calculated directly from the MC model. Five sets of intermolecular vectors, defined as 

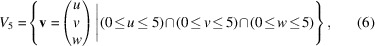

were included in all SRO calculations. This process was repeated ∼12 000 times starting from random A/B concentrations and random α_[001]_, α_[010]_, and α_[100]_ to create a pre-computed list of realistic SRO parameters that could be applied to any of the molecule pairs discussed above.

Although this MC method gave a good random selection of realistic SRO parameters, it neglected any symmetry constraints that would likely be present in real higher-symmetry systems. For example, in a hexagonal structure with a unique *c* axis, local ordering along [100] would likely be equal to that along [110]. We therefore supplemented the dataset with samples exhibiting such behaviour via an alternative approach. The values of the SRO parameters were defined using the damped oscillator function: 

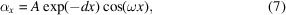

where *x* represents the set of symmetry-equivalent interatomic vectors, taking integer values from 1 denoting the nearest-neighbour shell to which the set of vectors belongs. *A* is the amplitude, *d* is a decay constant and ω is the frequency, with values varying randomly in the ranges 0.6–1.5, 0.1–1.2 and 0–1, respectively. These ranges were chosen to keep the maximum values of α between −1 and 1, and to ensure that they decayed to zero before the last shell of neighbours. The concentration of each molecule was held constant at 0.5. This produced a further 3864 sets of correlation parameters to use with molecules on hexagonal systems and 10 024 for tetragonal/cubic systems (equivalent to 14 additional disorder models per molecule pair).

We also considered the kind of experimental artefacts that could be present in real data. For example, reconstructed planes of diffuse scattering are not perfectly square and might contain dead zones from detector module gaps or masks. Such instrumental artefacts were extracted from five real single-crystal test datasets collected on BM01 at SNBL, ESRF. Each one was reconstructed in *Meerkat* (Simonov, 2019 ▸) on a 256 × 256 × 256 grid, and the areas where the scattering was equal to zero were extracted.

Having prepared lists of molecule pairs, SRO coefficients and artefacts, the complete training and validation datasets were generated using the following steps. For each molecule pair:

(1) A set of SRO parameters and A/B concentrations was randomly selected from the pre-computed list, and the lattice parameters of the model were recalculated based on the sizes of A and B, their respective concentrations and assuming Vegard’s law.
(2) Several *hkl* planes were selected. For the MC-generated SRO, 12 were selected – 4 along each axis. For the symmetry-restricted SRO, 4 were selected normal to the unique axis. See Section 1.4 of the supporting information for further details on how planes were selected.
(3) *I*_FF_(**Q**), *I*_SRO_(**Q**) and *I*_D_(**Q**) were calculated using equations (1), (3) and (5) for the selected planes. Grids of 256 × 256 pixels were used, as a compromise between resolution and data storage, with a *Q*_max_ of between 6 and 8 Å^−1^, corresponding to a typical data collection on BM01 at SNBL (Dyadkin *et al.*, 2016 ▸) at a wavelength of 0.7 Å.
(4) Stages (1)–(3) were repeated 28 times with different SRO parameters.
(5) A Wasserstein distance check (see Section 1.5 of the supporting information) was performed across the scattering planes generated. This examines the topological uniqueness of each sample and removes redundant planes, as too many such samples would result in our network overfitting and developing bias.
(6) The square roots of the remaining scattering planes were taken to emphasize low-intensity features, and they were each normalized between −1 and 1 (as opposed to normalizing by the most extreme samples). This normalization was performed to avoid any bias arising from scaling and had the benefit of not minimizing the importance of any individual sample owing to a smaller scaling factor.
(7) Each sample was multiplied by a randomly selected artefact plane. Regions where artefacts were present became the lower bound of −1, while everywhere else retained its previous intensity value.

The final training dataset thus comprised 1049 molecule pairs, each with 28 sets of SRO parameters. Two-hundred scattering planes were generated for each molecule pair (144 from MC-generated SRO models and 56 from symmetry-restricted models), totalling 209 800 data samples. Subsequently, 5.4% of samples were deemed not unique as a result of the Wasserstein distance check, reducing the total number of samples to 198 421. For the validation set, there were 98 molecule pairs, each with 12 MC-generated SRO models. Twelve scattering planes were calculated per model, giving 14 112 samples. This was reduced by the Wasserstein distance check, leaving 12 607 final validation samples.

### 2.2. Network construction

A Pix2PixGAN (Isola *et al.*, 2018 ▸) is based on a GAN, a class of deep-learning frameworks developed by Goodfellow *et al.* (2014 ▸). GANs comprise two neural networks: a discriminator, *D*, and a generator, *G*. The goal of *D* is to estimate the probability that any input sample belongs to the training data distribution, *y* ∼ *p*_d_(*y*). In other words, it is trained to correctly guess whether a given *I*_FF_(**Q**) sample is associated with the pool of *I*_FF_(**Q**) images. In contrast, *G* takes a latent variable *z* ∼ *p*_*z*_(*z*) and attempts to learn *p*_d_, *i.e.* the underlying features that describe *I*_FF_(**Q**), such that it can faithfully produce a new but realistic image. The two are trained in an adversarial manner, where *D* aims to label the training samples as real and those from *G* as fake, while *G* tries to trick the discriminator into believing its samples are real.

In a Pix2PixGAN, the generator has a U-Net architecture (Ronneberger *et al.*, 2015 ▸) (see Fig. 3 of the supporting information), and, in our case, takes scattering data, *x*, as input. It encodes this input into a low-dimensional latent space before decoding it into *y*_*i*_, where *i* can be the *I*_FF_(**Q**) or *I*_SRO_(**Q**) target data. Noise, *z*, is included through a 50% dropout rate in *G*. This approach follows Isola *et al.* (2018 ▸) and ensures the network captures inherent uncertainty in mapping between the input scattering and respective output domains, prevents overfitting to the training data, and encourages exploration to improve the diversity and quality of output. The discriminator is a standard CNN (see Fig. 4 of the supporting information), which takes as input either *y*_*i*_|*x* or *y*_*i*,GT_|*x*, with GT corresponding to the ground-truth target. In practice, *x* is concatenated to the input *y* along the channel dimension before being fed into the discriminator.

**Figure 2:**
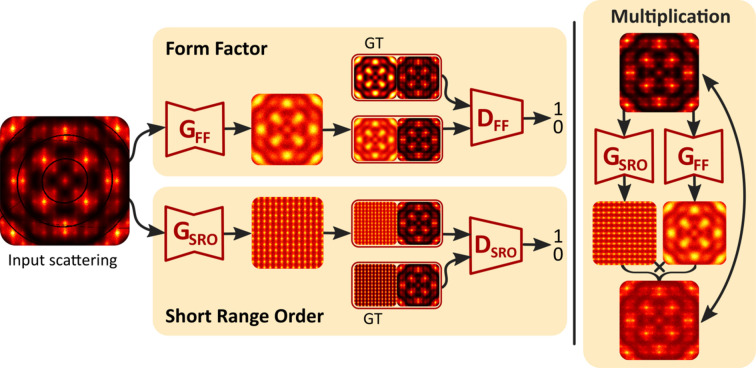
A schematic diagram of the network architecture (see Section 2 of the supporting information for more details). The discriminators were fed GT and generator output samples. They were trained to label the former as real and the latter as fake. Both inputs to the discriminators were paired with their respective clean scattering data (*i.e.* without the artefacts). The generators were trained adversarially, receiving the scattering data with artefacts as input into both *I*_FF_(**Q**) and *I*_SRO_(**Q**) U-Net generators, which encoded and decoded this into our desired output. After one full iteration of training for the discriminators and generators, an additional training stage occurred where the *I*_FF_(**Q**) and *I*_SRO_(**Q**) generated outputs were multiplied, and the error between this and the clean scattering data was used to train the generators.

We employed two Pix2PixGANs, one each for *I*_FF_(**Q**) and *I*_SRO_(**Q**), that were trained in parallel. At the end of each iteration, we included an additional training step that took the *I*_FF_(**Q**) and *I*_SRO_(**Q**) outputs from the two generators and multiplied them, with the goal of optimizing the product to match the input scattering data. Fig. 2 ▸ provides a schematic diagram for the whole training process, and specific architecture details are given in Section 2.1 of the supporting information.

Given that the goal of *G* is to minimize the probability that *D* classifies its samples as fake, conditioned on scattering input *x*, we applied the objective function used by Isola *et al.* (2018 ▸): 

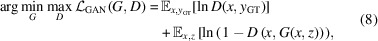

where *G*(*x*, *z*) represents generated output *y*_FF_ or *y*_SRO_. The term 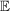 represents the expectation value. *G* in a Pix2PixGAN differs from a conventional GAN by also employing a pixel-wise L1 loss (equivalent to the pixel-wise mean error) between the GT and generated output: 

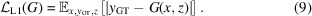

Thus, the loss for each of the parallel Pix2PixGANs can be written as 

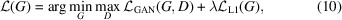

where we took λ as 100, following Isola *et al.* (2018 ▸). *G* and *D* had their weights and biases frozen when training their respective opponent. For the additional training stage that used the output from the *I*_FF_(**Q**) generator as auxiliary information to the optimization of the *I*_SRO_(**Q**) generator, and *vice versa*, we multiplied the output of *G*_SRO_ and *G*_FF_ and calculated the smooth L1 loss between this and the GT: 

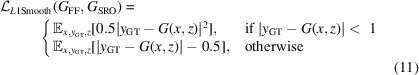

where 

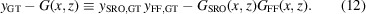

Due to the Cauchy–Schwarz inequality, the two outputs multiplied did not correspond exactly to the normalized input scattering data we fed into *D* and *G*. Therefore, we compared the output with the product of the normalized *I*_FF_(**Q**) and *I*_SRO_(**Q**) GTs. The smooth L1 loss combines the advantages of both L1 and L2 losses [L2 here referring to the pixel-wise mean squared error (MSE)]: we had steadier gradients when *y*_GT_ − *G*(*x*, *z*) was large and smaller oscillations in our model parameters when *y*_GT_ − *G*(*x*, *z*) was small.

We found that *D* could not distinguish between artefacts present in the scattering input and any still present in *y*_*i*_ owing to the mixing of *x* and *y* in the CNN if we used artefact-stained inputs to the discriminator. Consequently, *G* would never learn to remove them and would even add them when applied to clean examples. We, therefore, opted to use clean scattering inputs to *D*. This did not impact the final model for a general-use case where only *G* is required.

We tested some alternative architectures, including transformers (Vaswani *et al.*, 2017 ▸), different training strategies and loss functions over the course of the network development (more detail is given in Sections 2.2 and 2.3 of the supporting information), but found they were a detriment to overall network performance. Therefore, the following results were obtained through the network as described above.

We implemented the parallel Pix2PixGANs in Python using the *PyTorch* module (Paszke *et al.*, 2019 ▸) and trained both simultaneously on the 198 421 scattering planes for 200 epochs beyond loss convergence on a NVIDIA RTX 3090. The final models are available on GitHub (https://github.com/dclw29/DSFU-Net), including the trained generators, collectively referred to as DSFU-Net (diffuse scattering factorization U-Net) from hereon in, for users to extract their own *I*_FF_(**Q**) and *I*_SRO_(**Q**) data. There is also a pipeline script to perform the necessary pre-processing. To use DSFU-Net, prospective users need only prepare their input to be a size of 256 × 256 pixels.

## 3. Results and discussion

### 3.1. Validation dataset

**Figure 3:**
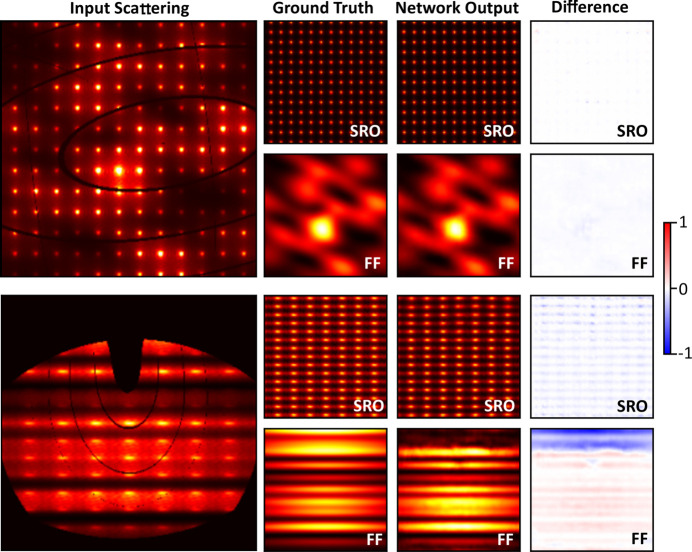
Two examples of scattering inputs from the validation dataset and their corresponding DSFU-Net outputs and GTs, representing one of the best performances (top) and one of the worst (bottom). Scattering planes are shown on a square-root scale to better emphasize low-intensity features.

The 12 607 unseen scattering planes reserved as the validation dataset were input into DSFU-Net, and the *I*_FF_(**Q**) and *I*_SRO_(**Q**) outputs were analysed to assess DSFU-Net’s performance. Fig. 3 ▸ shows two examples of input scattering planes, the generated outputs and the corresponding GTs. According to the mean squared differences between the outputs and the GTs, these two correspond to some of the best and worst examples.

In the first example, the visual match between the generated output and the GT is excellent; the network essentially completely reproduces the form factor and SRO components. The second example has areas where the decomposition is very good, but DSFU-Net struggles to recover the correct intensities in areas with low scattering. In this example, this is most noticeable at the top of the plane, which corresponds to a dead zone in the input scattering. While the general pattern of *I*_SRO_(**Q**) is still produced, the quality of the match to the GT becomes worse in this area, and DSFU-Net has trouble predicting any significant intensity for *I*_FF_(**Q**). However, for smaller gaps in the input scattering, such as the rounded corners and the elliptical lines, DSFU-Net is able to adeptly fill in these areas based on the surrounding context, demonstrating its robust understanding of the task derived from the training data.

To make a similar quantitative comparison over all 12 607 validation samples, we employed two commonly used metrics in the deep-learning community that measure the difference between two sets of distributions: the Fréchnet inception distance (FID) (Heusel *et al.*, 2018 ▸) and the kernel inception distance (KID) (Binkowski *et al.*, 2021 ▸) (see Section 3.1 of the supporting information for more details). We also used the more familiar pixel-wise MSE. FID and KID are singular values, while the MSE provided in Table 1 ▸ represents the average of all individual sample comparisons. For all three metrics, two identical distributions would return a value of zero, and, as an upper limit, we provide a baseline comparison between a dataset of uniform noise and the GT.

In all the metrics, the scores for *I*_SRO_(**Q**) and *I*_FF_(**Q**) are at least an order of magnitude smaller than the respective noise comparison. In terms of the general magnitude of the FID and KID scores, both *I*_SRO_(**Q**) and *I*_FF_(**Q**) are closely aligned with those from established benchmarks used to assess GANs in the computer-vision field (Betzalel *et al.*, 2022 ▸). The average MSE shows the same trend. Looking in more detail at the distribution of validation MSEs (see Fig. 6 of the supporting information), 90% of the DSFU-Net generated *I*_SRO_(**Q**) planes have an MSE of less than 0.032 compared with the GT. The *I*_FF_(**Q**) component performs even better, with 90% having an MSE of less than 0.014. These low scores, compared with noise, indicate that DSFU-Net has successfully learnt the underlying features of the data distribution and can map an input scattering plane to the desired factorized components.

The *I*_FF_(**Q**) scores are noticeably larger than *I*_SRO_(**Q**). This can be attributed to the greater diversity of intensity topology in these images and the fact that *I*_SRO_(**Q**) tends to have lower intensities. In the latter case, Fig. 6 of the supporting information demonstrates that the *I*_SRO_(**Q**) noise–GT comparison is much flatter, in some cases less than the noise–noise comparison, which could lead to an artificial decrease in the scores.

Finally, the training scores are marginally better than those for the validation. This fact is unsurprising as the validation set contains entirely new molecules leading to novel scattering examples never seen by DSFU-Net during training. However, these differences are very small, demonstrating that the network has generalized beyond the training set and is applicable to unseen examples.

### 3.2. Application to an experimental example

The next step was to benchmark DSFU-Net against a solved experimental example. One such case is the molecular crystal tris-*tert*-butyl-1,3,5-benzene tricarboxamide. The structure, solved from single-crystal data (Kristiansen *et al.*, 2009 ▸) (illustrations and coordinates are provided in Section 3.2 of the supporting information), consists of columns of molecules stacked along the *c* axis in one of two orientations. Molecule orientation is constant along each column, determined by a network of hydrogen bonds, but the columns have a negative nearest-neighbour correlation in the *ab* plane. This leads to a diffuse scattering pattern consisting of a hexagon surrounding each Bragg peak, modulated by the form factor difference between the two molecule orientations. The *I*_SRO_(**Q**) component was extracted analytically by Schmidt & Neder (2017 ▸). Since DSFU-Net requires no prior knowledge of the average structure, we used their equivalent method (dividing by the average form factor squared and projecting into a single Brillouin zone) as the benchmark. The *I*_FF_(**Q**) component can be calculated directly from the known disordered structure.

**Figure 4:**
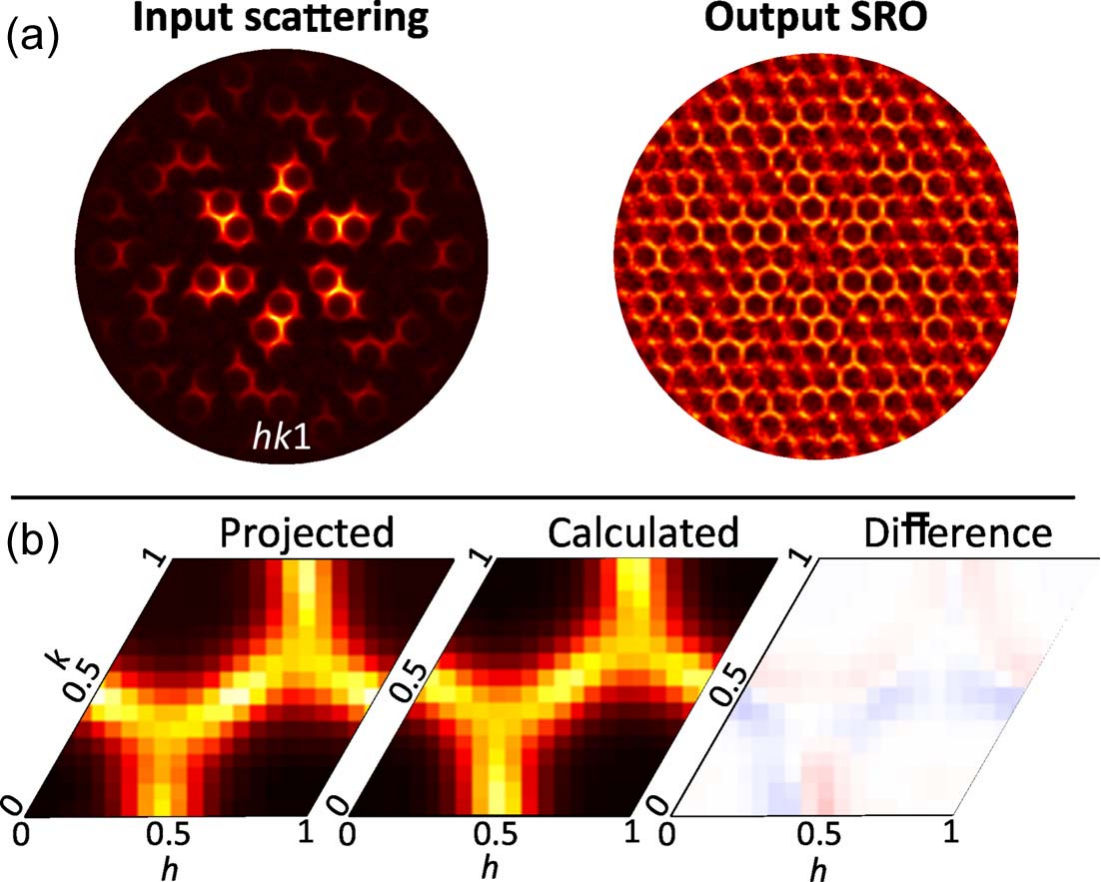
(*a*) The *hk*1 scattering plane from tris-*tert*-butyl-1,3,5-benzene tricarboxamide that was used as the DSFU-Net input, and the corresponding output *I*_SRO_(**Q**). (*b*) The DSFU-Net output projected into one reciprocal-space unit cell, a reciprocal unit cell calculated from the SRO parameters refined in this work, and the difference between them.

Experimental data for the *hk*1 scattering plane were obtained from Simonov *et al.* (2014*a* ▸) with kind permission, having had the Bragg peaks and background already removed (see note on the importance of this in Section 3.3 of the supporting information). The data were reconstructed on a square 256 × 256 pixel grid using the torchvision.resize method in Python, and input into DSFU-Net using the available pipeline, taking seconds to produce the outputs shown in Figs. 4 ▸(*a*) and 5 ▸(*a*).

#### 3.2.1. Short-range order

Fig. 4 ▸(*a*) shows the raw DSFU-Net *I*_SRO_(**Q**) output in the top right. It captures the expected honeycomb pattern, performing particularly well in regions with the highest input scattering intensity. Outside these regions, the pattern becomes noisy as DSFU-Net struggles to recapitulate the correct intensity owing to the low-intensity values, in agreement with the earlier performance assessment on the most challenging validation samples. With these experimental data, low intensities imply that the signal-to-noise ratio decreases. Therefore, while DSFU-Net can ignore some small fluctuations in intensity and indeed ‘fill in’ blank regions, the output is noisier than it would be for a clean input (see Fig. 8 of the supporting information for comparison). This could potentially be improved by adding statistical noise to the training data.

Following the method of Schmidt & Neder (2017 ▸), quantitative SRO parameters can be obtained from this output by projecting it into a single reciprocal-space unit cell. To minimize the effect of noise from low intensity, regions where the intensity is below 5% were excluded and sixfold rotation symmetry was applied. The result is shown in Fig. 4 ▸(*b*) and looks qualitatively very similar to that obtained by Schmidt & Neder (2017 ▸). The cosine series in equation (3) multiplied by a scale factor was fitted to this projected reciprocal unit cell using a linear least-squares refinement. Since the scattering pattern has sixfold rotation symmetry, we applied restrictions to the SRO parameters, for example, α_[100]_ = α_[110]_ = α_[010]_ *etc.*, and, given that there is no disorder along the *c* axis, we excluded any vector where *v*_*z*_ ≠ 0 from the refinement.

The refined values are listed in Table 2 ▸ compared with those obtained by Schmidt & Neder (2017 ▸) using two established methods: (1) refinement of the 3D-ΔPDF in the *Yell* program and (2) a least-squares refinement against the *I*_SRO_(**Q**) extracted through the division of the scattering data by the average form factor squared (Schmidt & Neder, 2017 ▸).

The refined SRO parameters provide an *I*_SRO_(**Q**) that is a great fit to the projected intensities, as demonstrated by the difference map in Fig. 4 ▸(*b*). Therefore, these parameters are a reasonable model for the disorder in this material. They are also consistent with those obtained through the established method from Schmidt & Neder (2017 ▸). While there are some minor discrepancies, we are able to reproduce the relative magnitudes and, crucially, the correct signs. Comparative results refined without any pre-processing are shown in Section 3.3 of the supporting information and corroborate this finding. This result demonstrates that the DSFU-Net output is of sufficiently high quality that it could be used for quantitative analysis.

#### 3.2.2. Form factor

**Figure 5:**
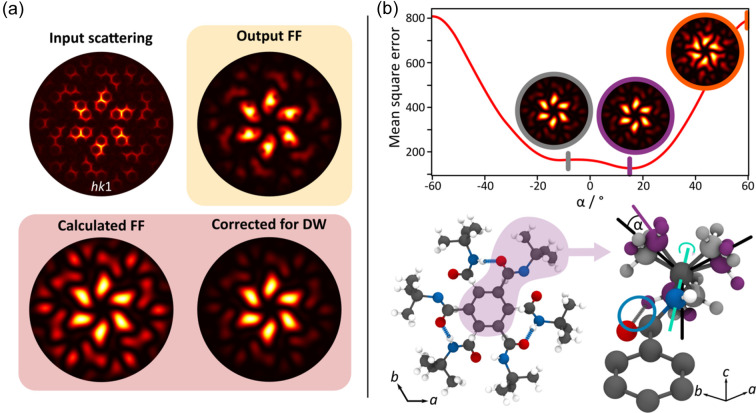
(*a*) The *hk*1 scattering plane from tris-*tert*-butyl-1,3,5-benzene tricarboxamide that was used as the DSFU-Net input, and the corresponding output *I*_FF_(**Q**). Below is the *I*_FF_(**Q**) calculated from the known molecular structures (given in Table 5 of the supporting information) and the same calculated pattern corrected by the DW factor to make it consistent with the experimental scattering. (*b*) A plot of the mean square error between the DSFU-Net output *I*_FF_(**Q**) and the calculated *I*_FF_(**Q**) as the *tert*-butyl group is rotated about the N—C bond. Insets show the calculated *I*_FF_(**Q**) at different angles. Below, the published average structure of tris-*tert*-butyl-1,3,5-benzene tricarboxamide, with carbon, oxygen, nitrogen and hydrogen atoms shown in grey, red, blue and white, respectively. The hydrogen-bond interactions between the amide hydrogens and oxygen in the molecule are stacked above. A zoomed section highlighting the two *tert*-butyl orientations at each of the minima (palatinate purple and grey), the rotation axis (cyan line), and the formation of an additional hydrogen-bond-like interaction between an oxygen atom and a methyl hydrogen for α = 16° (circled in blue) is shown on the right. The black lines show the orientation chosen to have α = 0°.

The network output *I*_FF_(**Q**) is shown in Fig. 5 ▸(*a*). The similarities between the input and output are clear, and DSFU-Net appears to have captured the key features. The *I*_FF_(**Q**) calculated from the published structure is shown in the bottom left. Relative to this, the output *I*_FF_(**Q**) accurately reproduces the positions of the main features very well; however, there are some areas where the intensities are not correctly predicted. Firstly, the circular regions of low intensity that appear as small holes in the inner part of the pattern. This is probably another manifestation of the inherent limitations in regions of very low scattering intensity.

The other noticeable difference is that the overall intensities of the DSFU-Net output appear to decay faster with *Q*. On simulated scattering from tris-*tert*-butyl-1,3,5-benzene tricarboxamide, calculated using equation (5), DSFU-Net was able to reproduce the two components almost exactly (see Section 3.4 of the supporting information, revealing that this additional *Q* dependence is a feature of the experimental data and not a limitation with the network.

The source of this discrepancy is the assumption that we can neglect all displacive disorder in the crystal. A more complete description of the diffuse scattering is given by 

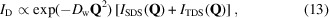

where *D*_w_ is the Debye–Waller factor (DW); *I*_SDS_ is the diffuse scattering from static disorder, encompassing both static displacements and chemical SRO; and *I*_TDS_ is the thermal diffuse scattering arising from dynamic structural displacements (Mezger *et al.*, 2006 ▸). In this material, static displacements are expected to be negligible as both molecular orientations are the same size. Most TDS comes from atomic motions associated with the acoustic phonons and appears very close to the Bragg peaks, which, in this case, were only a few pixels wide and were removed during pre-processing. The DW factor is therefore expected to account for a large part of the inconsistency between the DSFU-Net *I*_FF_(**Q**) and the calculated one. A suitable correction could therefore be applied to the calculated *I*_FF_(**Q**) by multiplying by the exponential term in equation (11). *D*_w_ could potentially be calculated from the average structure or even refined against the DSFU-Net output *I*_FF_(**Q**). However, a refined value may not correspond to the experimentally determined thermal parameters as it will be sensitive to any other displacive disorder that may be present. Nevertheless, this method was used to generate the DW-corrected image in the lower right corner of Fig. 5 ▸(*a*), significantly improving the match between the calculated *I*_FF_(**Q**) and the DSFU-Net output.

Using this DW correction, the DSFU-Net output *I*_FF_(**Q**) could be applied to discriminate between two similar structural models, comparable to the analysis carried out by Chodkiewiez *et al.* (2016 ▸). As an example, consider the orientation of the *tert*-butyl groups. Fig. 5 ▸(*b*) shows the known structure of tris-*tert*-butyl-1,3,5-benzene tricarboxamide viewed down the *c* axis and a zoom of one of the *tert*-butyl groups, with the grey carbon atoms indicating the position of the *tert*-butyl in the published structure. Fig. 5 ▸(*b*) also explores *I*_FF_(**Q**) as a function of rotation angle around the C—N bond, shown as the cyan line in the zoomed structure, to assess whether it would be possible to refine the angle using the network output. For each angle, the mean square error between the network output *I*_FF_(**Q**) and the calculated *I*_FF_(**Q**) was found and is plotted in red. The result varies smoothly with angle from a fairly flat and wide minimum centred at 0° up to maxima at ±60°. The published structure, shown as the inset ringed in grey, sits comfortably within this minimum at −7°, while the maximum at 60° corresponds to the inset ringed in orange. Comparing the two, it is immediately apparent that the published structure provides a better match to the DSFU-Net output, and the molecule probably prefers this orientation to avoid steric crowding between the oxygen atom and one of the methyl carbons.

We find that between −20 and 20°, the *I*_FF_(**Q**) is very similar, yet the MSEs show two minima. The corresponding *tert*-butyl orientations are overlaid in the zoomed structure in Fig. 5 ▸(*b*) in grey and palatinate purple. The first minimum, at −11°, is very close to the published structure, demonstrating the suitability of the network output to be used in structural refinements. The structure corresponding to the second, slightly deeper, minimum at 16° has one of the methyl hydrogens 2.0 Å away from the oxygen atom, indicating the possibility of a hydrogen-bond-like interaction [highlighted in Fig. 5 ▸(*b*) by the blue circle]. While we cannot say for certain that this double minimum is real and not just stochastic variations in a flat landscape, the presence of this potential hydrogen bond seems chemically plausible. Regardless, this quantitative use of the DSFU-Net output *I*_FF_(**Q**) shows that it can be a valuable source of structural information, one that is not readily available by any existing method. Given the sensitivity of *I*_FF_(**Q**) to small changes in molecular structure, it could be used to elucidate more precise chemical structures than Bragg diffraction alone.

### 3.3. Limitations

DSFU-Net will produce two components from any input, providing that the size is correct. Since it was trained to separate periodic sharp patterns from continuous ones, it will, for example, separate Bragg peaks from a smooth background. However, the output components will only be meaningful if the assumptions and mathematics underpinning the training data can be applied to input scattering data. To be able to interpret the output as the scattering due to chemical SRO and molecular form factors, the system in question must have binary substitutional disorder with one disordered site per unit cell and, for the best quantitative results, disorder from displacement and phonons should be negligible. Tris-*tert*-butyl-1,3,5-benzene tricarboxamide is known to have a small size-effect relaxation (Simonov *et al.*, 2014*a* ▸); therefore, small deviations from these assumptions can be tolerated. However, as displacive disorder becomes more prevalent, the resulting DSFU-Net *I*_FF_(**Q**) may be different from any calculated ones as it will also be sensitive to any internal molecular distortions arising from the relaxations or other such displacements.

## 4. Conclusions

We have designed a deep-learning method, DSFU-Net, based on the Pix2PixGAN (Isola *et al.*, 2018 ▸), which takes as input a plane of diffuse scattering and separates the contributions from the molecular form factor and the chemical short-range order, facilitating local structure modelling. DSFU-Net was trained on 198 421 samples of simulated scattering data and performed extremely well on 12 607 simulated validation datasets, with >90% of outputs having a mean square error of <0.04, relative to the ground truth, even when the inputs included dead zones from detector gaps. We have demonstrated that DSFU-Net also generalizes to real experimental data, and that the output *I*_FF_(**Q**) and *I*_SRO_(**Q**) are both of sufficient quality that refinement of disorder parameters or structural elements is possible. A key success is that DSFU-Net offers a means of extracting the form factor directly, without requiring understanding of either the average structure or the short-range order.

This method builds on the diffuse scattering analysis techniques implemented by Schmidt & Neder (2017 ▸) and Chodkiewiez *et al.* (2016 ▸) by providing direct access to both the form-factor and short-range-order components for single-crystal systems exhibiting pure binary substitutional disorder. Our neural-network approach requires minimal prior knowledge of the system, negates the need for difficult division owing to mathematical instabilities when dividing by regions of low intensity and is able to compensate for small regions with missing data. It takes seconds to obtain the separate components, providing immediate qualitative understanding and facilitating an improved starting point for more complex modelling. As such, DSFU-Net would be well suited to being integrated as part of an automated pipeline, for example, on a beamline, to direct decision making in real time.

For a more quantitative description of disorder, the Warren–Cowley SRO parameters can be extracted directly from *I*_SRO_(**Q**) using least-squares refinement. Visual inspection of *I*_FF_(**Q**) can be used to distinguish between similar models, helping to uncover the disordered components of the structure. Since *I*_FF_(**Q**) is very sensitive to structure, perhaps more so than Bragg data, our network output can also be used to refine structural details, such as the relative orientations of molecules A and B or, as exemplified here, the position of functional groups. In the special case where *F*_B_ is zero (*i.e.* B is a vacancy), the structure of A could potentially be solved by phasing the network output *I*_FF_(**Q**) directly, similar to the analysis of Simonov *et al.* (2017 ▸). This opens up possibilities of structure solution using only diffuse scattering, an attractive option considering continuous scattering does not suffer from the phase problem (Ayyer *et al.*, 2018 ▸), and it could be particularly beneficial if crystallinity or crystal size is an issue.

DSFU-Net is available on GitHub (https://github.com/dclw29/DSFU-Net) with documentation and scripts allowing users to apply the trained neural networks to their own problems. If required, the applicability of DSFU-Net could be easily extended to other systems with one disordered site per unit cell. For example, those containing pure displacement disorder or potentially a mixture of displacement and substitutional disorder, given an appropriate training dataset. Our GitHub provides the means to generate new training data and retrain the neural network. However, expansion to more complex systems is beyond the scope of the existing architecture.

As deep-learning techniques are continually improving and their use becoming more widespread, it is evident that they will become increasingly relevant to solving long-standing problems within crystallography. In this work, we have demonstrated, to the best of our knowledge, the first application of generative deep learning to disentangle components of diffuse scattering data. Our work sets the foundation for the use of deep learning as a tool to tackle more complex problems within the field, such as the inclusion of displacement disorder, ultimately working towards a general and automated workflow for the analysis of single-crystal diffuse scattering.

## 5. Related literature

The following references are only cited in the supporting information for this article: Deng *et al.* (2009 ▸), Gulrajani *et al.* (2017 ▸), Naderi *et al.* (2022 ▸), Saxena & Cao (2023 ▸), Silva (2018 ▸), Simonyan & Zisserman (2015 ▸) and Virtanen (2020 ▸). 

## Supplementary Material

Supporting information containing additional details on methods, analyses and benchmarking. DOI: 10.1107/S2052252523009521/fs5226sup1.pdf

## Figures and Tables

**Table 1:** FID, KID and MSE between the DSFU-Net-generated and GT scattering planes for the validation (12 607 total samples) and training datasets (random selection of 50 000 samples) compared with a dataset of uniform noise versus the validation GT as a baseline

		Validation		Training
		Noise versus GT	Generated versus GT	Generated versus GT
*I*_SRO_(**Q**)	FID	372	13.1	8.35
	KID	0.741	0.003	0.002
	MSE	0.951	0.015	0.010
*I*_FF_(**Q**)	FID	464	36.9	33.3
	KID	0.761	0.008	0.018
	MSE	1.00	0.006	0.004

**Table 2:** The SRO parameters refined by Schmidt & Neder (2017 ▸) using (1) *Yell* and (2) a linear least-squares refinement following division by the average form factor squared, compared with (3) the least-squares refinement following the application of DSFU-Net

**v** †	*Yell*	Division by |*f*_average_|^2^	DSFU-Net
(1,0,0)	−0.2516	−0.2212	−0.2474
(2,0,0)	0.0984	0.1042	0.1094
(2,1,0)	0.0950	0.1033	0.0824
(3,0,0)	−0.0345	−0.0218	−0.03075
(3,1,0)	−0.0532	−0.0330	−0.0529
(3,2,0)	−0.0435	−0.0394	−0.0378
(4,0,0)	0.0164	0.0156	0.0109
(4,1,0)	0.0256	0.0211	0.0186
(4,2,0)	0.0310	0.0332	0.0252
(4,3,0)	0.0165	0.0225	0.0107
(5,0,0)	−0.0090	−0.0044	−0.0026
(5,1,0)	−0.0128	−0.0046	−0.0055
(5,2,0)	−0.0175	−0.0148	−0.0104
(5,3,0)	−0.0149	−0.0110	−0.0086
(5,4,0)	−0.0073	−0.0069	−0.0037

† **v** is given here in reciprocal lattice units.
