# Supplementary material for: Unravelling the components of diffuse scattering using deep learning
Source: IUCrJ. 2024 Jan 1;11(Pt 1):34–44. doi: 10.1107/S2052252523009521 (PMC10833394; doi:10.1107/S2052252523009521)
Supplement: Supplementary file 1 [file m-11-00034-sup1.pdf]

IUCrJ

Volume 11 (2024)

Supporting information for article:

Unravelling the components of diffuse scattering using deep learning

Chloe A. Fuller and Lucas S. P. Rudden

Unravelling the components of diffuse scattering using deep learning

Chloe A. Fuller and Lucas S. P. Rudden

Supplementary Information

1 Compiling the dataset

1.1 Molecule pairs

For each molecule in the list, the approximate centre of mass was positioned at the origin, and the molecule was rotated such that major axes lay along the Cartesian axes. Molecules were assigned a lattice type, based on the shape/anisotropy of the molecule. Single atoms and molecules which were roughly spherical (e.g. tetrahedra, octahedra etc), were assigned a primitive cubic lattice. Molecules in which one axis was significantly different from the other two were assigned a tetragonal lattice, with the unique axis being either short or long and pointing along b. Molecules based on benzene rings were assigned a hexagonal lattice, with the unique axis also as b.

Next, each molecule was given values for lattice constants. For the single atoms, the literature value for the cubic primitive lattice of the metal was used. For the others, the lattice parameters were set equal to the longest interatomic distance in the molecule along that direction +1.5 Å. If the terminating atoms were F or O, additional distances of 2 or 2.5 were used instead of 1.5. For all molecules with non-cubic lattices, the unique axis was always b, and $a = c$. This process of cell parameter assignment is largely arbitrary and we have excluded monoclinic/triclinic lattices. For the purpose of training the network, the lattice parameters do not need to be exact, they just need to be different enough to produce a change in the position of the maxima. Likewise, we do not necessarily need to include all Bravais lattices and restricting the choice means that molecules can be grouped more easily. Additional lattices can always be added to the training dataset at a later date if it proves necessary.

To expand the dataset further, some of the molecules can be rotated to form unique orientations. A molecule can be paired with a rotated version of itself. For cubic structures (excluding the spherically symmetric single atoms), three random angles (10-340°) were generated, and a rotation matrix was applied using these angles, using the Cartesian axes as a basis. If the angles happen to produce a rotation that is symmetrically equivalent to the original molecule, this was discarded and the process repeated. For the tetragonal and hexagonal molecules, the molecules were predominately rotated around the b-axis (to avoid having to change the cell parameters). A random rotation between 10 and 340° was chosen for this and a rotation of between -20 and 20° was applied around the other axes.

Molecules were allowed to pair only with other molecules with the same lattice type. For each molecule pair, a new set of lattice parameters is calculated based on the concentrations and lattice parameters of the individual molecules, assuming Vegard's law.

Table 1: Summary of the molecules comprising the training and validation datasets.

Group Number	Lattice	Molecules	Combinations
Training set			
0	Cubic	16	120
1	Tetragonal, long b	20	190
2	Tetragonal, short b	30	435
3	Hexagonal	24	276
4	Cubic/tetragonal extended structures	8	28
		98	1049
Validation set			
0	Cubic	5	10
1	Tetragonal, long b	6	15
2	Tetragonal, short b	8	28
3	Hexagonal	10	45
		29	98

The number of combinations denotes the number of unique pairs of molecules (i.e. AB = BA). The training dataset, therefore, contains a total of 1049 pairs of molecules, and the validation set 98 pairs (about 10%).

1.2 Monte Carlo simulations

Chemical short-range order models were constructed on a primitive cubic lattice with one pseudo-atom in each unit cell, represented by a spin (1 or -1), as shown in Figure 1.2.

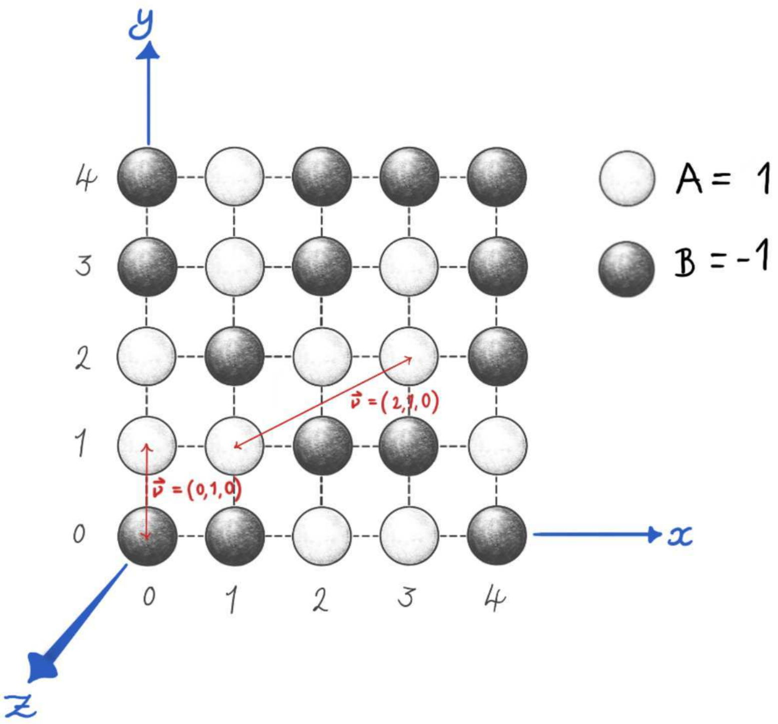

Figure 1: Schematic of a Monte Carlo chemical short-range order model. White and grey spheres represent spins of 1 or -1, respectively, which are numbered according to the unit cell in which they reside. The Cartesian axes are shown in blue and some interatomic vectors are shown in red.

The spins were added to the lattice randomly, then were ordered in 3D using a Monte Carlo algorithm. For each MC move, two opposite spins are selected at random and their values are swapped. The energy of the system before and after is calculated using an Ising energy:

$$E = \sum_{n=1}^{n=6} -k_{ij}\sigma_i\sigma_j \tag{1}$$

where k_{ij} is interaction energy between spins i and j and σ is the value of those spins (1/-1). The sum runs over the neighbours, n, of spin i. In this case, we included 6 neighbours: those along the unit cell vectors in the positive and negative directions. MC moves were accepted if a random number is less than

$$\exp(-\Delta E/T) - 0.5 \tag{2}$$

where ΔE is the difference in energy between the two states, T is the MC temperature (set at 0.1, as this was found empirically to give the best results). The minus 0.5 is used such that states with 0 change in energy have a 50% chance of being accepted, assisting in preventing the system from getting stuck in local minima.

After a certain number of MC moves (usually the number of atoms in the simulation box), the program has completed 1 MC cycle. At this point, the interaction energies are updated in order to drive the system towards the desired correlation.

$$k_{\text{new}} = k_{\text{old}} + (C_{\text{current}} - C_{\text{target}}) \times 100 \tag{3}$$

The program saves the configuration of the lattice after each cycle and the one that is closest to the target correlations is saved as the final configuration. Using this final configuration, the Warren-Cowley SRO parameters for all vectors up to and including [7,7,7] were calculated.

This whole process was run in a loop to create ~12000 lists of SRO parameters. For each one, the concentrations of the two molecules, m_{A} and m_{B}, were selected randomly between 0.1 and 0.5 (to avoid very low defect concentrations which make correlated configurations harder to generate) and the target correlations along 100, 010 and 001 were selected randomly from values between $(-m_{\text{A}}/(1-m_{\text{A}})$ (which is the lower correlation limit) and 0.5. These limits were chosen to provide the widest range of correlations, without becoming entirely long-range order. For systems with the higher correlations, the configuration was started from a fully ordered arrangement as the MC algorithm struggled to achieve this when initialised from a random state.

1.3 Interatomic vector sets

The number of interatomic vectors included in the calculation of $I_{\text{SRO}}(\mathbf{Q})$ has a large effect on the appearance of the diffuse scattering, as illustrated by Figure 2a. Ideally, the calculation would include all vectors extending to infinity and, in general, including more vectors improves the calculation. This is especially important with highly correlated systems, where $I_{\text{SRO}}(\mathbf{Q})$ resembles sharp peaks. Vectors are usually considered in sets corresponding to nearest-neighbour shells surrounding a central atom. To enable generalisation between different lattice shapes, we opted to define the first vector set as

$$V_n = \left\{ \vec{v} = \begin{pmatrix} u \\ v \\ w \end{pmatrix} \middle| (0 \le u \le n) \cap (0 \le v \le n) \cap (0 \le w \le n) \right\}. \tag{4}$$

A small benchmark was performed to decide how many sets of vectors to include in the $I_{\text{SRO}}(\mathbf{Q})$ calculations. We needed to include enough so that patterns would look realistic, but

including more vectors is computationally expensive, and the number of vectors in each set increases very quickly.

The diffuse scattering of molecule pair cyclohexane-dioxane was calculated for 50 different sets of SRO parameters, using V_n where $n = 1-7$. The difference in calculated scattering between successive vector sets was recorded and is plotted in Figure 2. The first 4 sets create large differences in scattering which then start to converge. The total number of vectors increases rapidly with each new shell and the marginal gain after $n = 5$ was not worth the huge increase in computational cost. Therefore, 5 neighbour sets were used in all calculations.

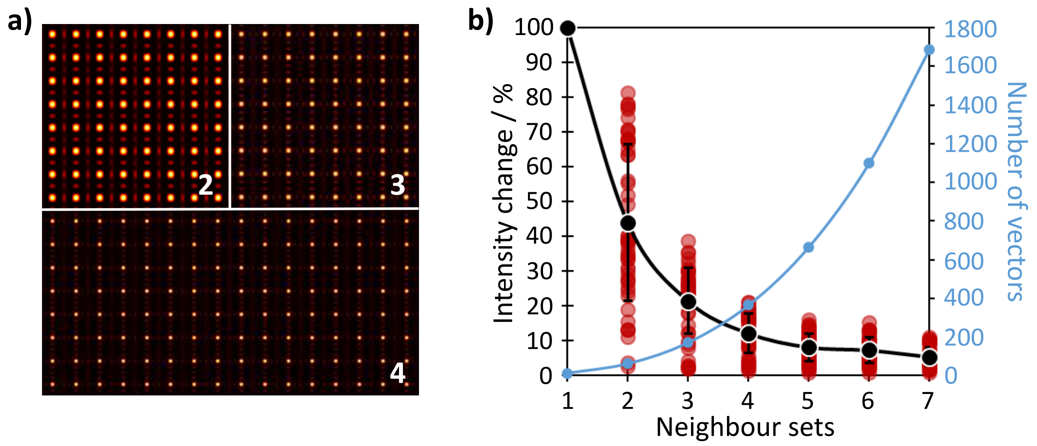

Figure 2: a) $I_{\mathrm{SRO}}(\mathbf{Q})$, calculated with 2,3 and 4 sets of neighbours. b) Total difference in intensity of the $I_{\mathrm{D}}(\mathbf{Q})$ of a cyclohexane-dioxane system as a function of neighbour sets included. Red points show the spread in the data across 50 different sets of SRO parameters, black shows the average and the error bar show the standard deviations. The blue line is plotted on the secondary y-axis and shows the number of vectors that need to be included with each set.

1.4 Selection of scattering planes to calculate

Due to the necessity of the large training dataset, the most relevant scattering planes for each model needed to be found in an automated way. We chose to calculate 12 scattering planes per model based on a compromise between extracting as many planes as possible while ensuring each one is unique in order to avoid problems with overfitting the network (12 referring to 4 planes from each of the x, y and z directions used). We implemented the following method for selecting scattering planes.

Since $I_{\mathrm{FF}}(\mathbf{Q})$ is continuous in reciprocal space, the most relevant planes will be where there are maxima in $I_{\mathrm{SRO}}(\mathbf{Q})$. The position of the maxima along a particular direction depends on the underlying correlations along the corresponding directions in the direct-space unit cell. Broadly speaking, positive correlations produce maxima underneath the Bragg peaks, negative correlations produce maxima somewhere between the Bragg peaks, and zero correlations give rise to constant intensities. Given that the b-axis is unique in most of our models, we started by calculating the 4 hxl planes. If the SRO parameter is positive, or close to 0 along b, we calculated $I_{\mathrm{D}}(\mathbf{Q})$ at the Bragg positions, corresponding to $h0l, h1l, h2l$ and $h3l$ scattering planes.

If the correlation along b is negative, it is more difficult to determine where the diffuse maxima will be as it depends on the concentration and correlations across multiple neighbour shells (unless in a 50:50 mixture). For this reason, we calculated all the planes between $h0l$ and $h1l$ (with a resolution of $(2 \times 8)/256$ Å^{-1}), and found the one with the most scattering by simply summing the total intensity in each plane. The offset in k relative to $k = 0$, o, is recorded and

the 4 final planes were calculated at hol , $h1{+}ol$, etc.

For the samples with symmetry-restricted SRO, only these 4 planes are used as ones taken along the other axes will not have the desired symmetry. For the rest of the samples, with MC-generated SRO, the first of those 4 planes was used to select the planes normal to the other two axes. The scattering is summed along the x and z directions to produce a 1D distribution. Scipy's peak finding algorithm [1] is then used to locate the areas with the 4 highest intensities. Peak finding was used instead of just taking the 4 highest values to ensure they can from separate areas of reciprocal-space to ensure uniqueness. The highest peaks were defined based on their prominence (instead of height). Using the peak positions, full xkl and hkx scattering planes are calculated. The 12 planes (3 along each direction) are stacked to create an array of size $256 \times 256 \times 12$, which is saved.

While this method does not guarantee the best planes, or even unique ones, it is reproducible. Selected planes then undergo additional checks for uniqueness through a Wasserstein Distance check.

1.5 Wasserstein Distance check

The Wasserstein Distance, or Earth Mover's distance, is a means of measuring the distance between two probability distributions. It can be interpreted as the energy cost of moving one probability distribution into the shape of another. If $f(x)$ and $g(x)$ are two probability distributions, let F and G define their cumulative distribution functions. The Wasserstein Distance between two distributions (formally the 2-Wasserstein Distance), W_{D}, is defined as:

$$W_{\mathrm{D}} = \left[\int_0^1 |F^{-1}(y) - G^{-1}(y)|^2 \mathrm{d}y\right]^{1/2}, \tag{5}$$

where F^{-1} and G^{-1} denote the respective quantile functions of F and G.

We reasoned that for a sample to be considered unique with respect to all other samples in its set, where a set is given by one molecule pair, its W_{D} between every other possible sample in the set should be greater than the W_{D} between two uniformly random distributions of the same dimensions. We ran 20000 total simulations of moving between two such distributions and converged on a W_{D} of 6.7. Thus, for each generated set of data, each sample is only accepted if its W_{D} is greater than 6.7 when compared with all other samples in the respective set. If it is less than this amount, then the first sample is accepted and the remaining discarded. This process removed roughly 5% of all samples from the initial dataset generation.

2 Network details

DSFU-net is based on a Pix2PixGAN [2], which has demonstrated proficiency in extracting and transferring underlying features present from one domain to another, such as converting satellite imagery to road maps. We experimented with several flavours of Pix2PixGANs, as discussed below, but found a relatively simple setup sufficed for our purposes. We first describe our network architecture before discussing architectures and approaches that were suboptimal.

2.1 Architecture details

Input to DSFU-net must always be $1 \times 256 \times 256$, the 1 referring to the RGB channel - i.e. greyscale images. In principle, an inspired user can take our GAN and retrain on larger images provided a decent training dataset, but this demands both increased computational resources and risks introducing known GAN training failure modes[3]. Interpolating from 512×512 or even larger to the required input of 256×256, while not providing as high a quality output,

should ultimately still provide quantitatively useful output provided one is careful with the interpolation process.

The details of each operation performed within DSFU-net are provided in Tables 2 and 3, while the corresponding schematics for both the generator and discriminator are given in Figures 3 and 4 respectively.

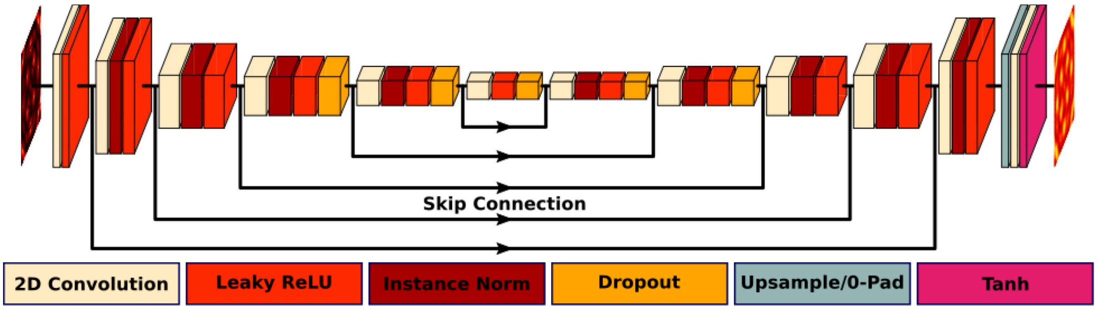

Figure 3: U-NET generator schematic with a $I_{\mathrm{FF}}(\mathbf{Q})$ example. Each layer is coloured according to its operation. Note the skip connections, where the output from the down blocks are concatenated with respective input to up blocks. The full details of the parameters for each layer are given in Table 2.

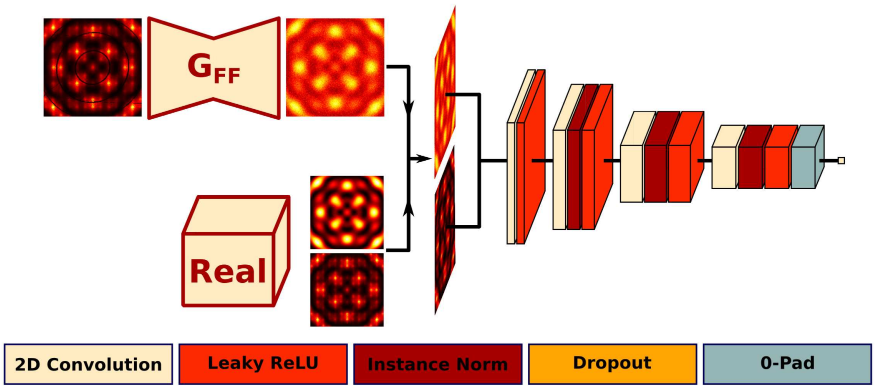

Figure 4: Discriminator schematic with a $I_{\mathrm{FF}}(\mathbf{Q})$ example. Each layer is coloured according to its operation. The full details of the parameters for each layer are given in Table 3.

Table 2: Summary of full U-NET generator architecture

Block	Operation	Parameters	Output Size
Input	-	-	$1 \times 256 \times 256$
Down 0	Convolution	Kernel=4, Stride=2, Padding=1, bias=False	$64 \times 128 \times 128$
	Leaky ReLU	Slope=0.2	
Down 1	Convolution	Kernel=4, Stride=2, Padding=1, bias=False	$128 \times 64 \times 64$
	InstanceNorm	Num. Features=128	
	LeakyReLU	Slope=0.2	
Down 2	Convolution	Kernel=4, Stride=2, Padding=1, bias=False	$256 \times 32 \times 32$
	InstanceNorm	Num. Features=256	
	LeakyReLU	Slope=0.2	
Down 3	Convolution	Kernel=4, Stride=2, Padding=1, bias=False	$512 \times 16 \times 16$
	InstanceNorm	Num. Features=512	
	LeakyReLU	Slope=0.2	
	Dropout	Probability=0.5	
Down 4	Convolution	Kernel=4, Stride=2, Padding=1, bias=False	$512 \times 8 \times 8$
	InstanceNorm	Num. Features=512	
	LeakyReLU	Slope=0.2	
	Dropout	Probability=0.5	
Down 5	Convolution	Kernel=4, Stride=2, Padding=1, bias=False	$512 \times 4 \times 4$
	LeakyReLU	Slope=0.2	
	Dropout	Probability=0.5	
Up 0	Transposed Convolution	Kernel=4, Stride=2, Padding=1, bias=False	$512 \times 8 \times 8$
	InstanceNorm	Num. Features=512	
	LeakyReLU	Slope=0.2	
	Dropout	Probability=0.5	
	Concatenate	Dimension=1	$1024 \times 8 \times 8$
Up 1	Transposed Convolution	Kernel=4, Stride=2, Padding=1, bias=False	$512 \times 16 \times 16$
	InstanceNorm	Num. Features=512	
	LeakyReLU	Slope=0.2	
	Dropout	Probability=0.5	
	Concatenate	Dimension=1	$1024 \times 16 \times 16$
Up 2	Transposed Convolution	Kernel=4, Stride=2, Padding=1, bias=False	$256 \times 32 \times 32$
	InstanceNorm	Num. Features=256	
	LeakyReLU	Slope=0.2	
	Concatenate	Dimension=1	$512 \times 32 \times 32$
Up 3	Transposed Convolution	Kernel=4, Stride=2, Padding=1, bias=False	$128 \times 64 \times 64$
	InstanceNorm	Num. Features=128	
	LeakyReLU	Slope=0.2	
	Concatenate	Dimension=1	$256 \times 64 \times 64$
Up 4	Transposed Convolution	Kernel=4, Stride=2, Padding=1, bias=False	$64 \times 128 \times 128$
	InstanceNorm	Num. Features=64	
	LeakyReLU	Slope=0.2	
	Concatenate	Dimension=1	$128 \times 128 \times 128$
Up 5	Upsample	Scale Factor=2	$128 \times 256 \times 256$
	ZeroPad	Padding=(1, 0, 1, 0)	$128 \times 257 \times 257$
	Convolution	Kernel=4, Stride=1, Padding=1, bias=True	$1 \times 256 \times 256$
	Tanh		

Table 3: Summary of full discriminator architecture

Block	Operation	Parameters	Output Size
Input	-	-	$2 \times 256 \times 256$
Block 0	Convolution	Kernel=4, Stride=2, Padding=1, bias=True	$64 \times 128 \times 128$
	LeakyReLU	Slope=0.2	
Block 1	Convolution	Kernel=4, Stride=2, Padding=1, bias=True	$128 \times 64 \times 64$
	InstanceNorm	Num. Features=128	
	LeakyReLU	Slope=0.2	
Block 2	Convolution	Kernel=4, Stride=2, Padding=1, bias=True	$256 \times 32 \times 32$
	InstanceNorm	Num. Features=256	
	LeakyReLU	Slope=0.2	
Block 3	Convolution	Kernel=4, Stride=2, Padding=1, bias=True	$512 \times 16 \times 16$
	InstanceNorm	Num. Features=512	
	LeakyReLU	Slope=0.2	
Block 4	ZeroPad	Padding=(1, 0, 1, 0)	$512 \times 17 \times 17$
	Convolution	Kernel=4, Stride=1, Padding=1, bias=False	$1 \times 16 \times 16$

We settled on a latent space of 4×4 within the U-NET generator as a compromise between avoiding overfitting and ensuring we capture the essential features necessary to map between our sets of domains. Alternative latent spaces, such as 2×2 and 8×8 gave higher losses overall, resulting in slightly worse quality output in terms of the FID and KID scores.

2.2 Alternative architectures

Transformers [4] are able to focus attention on regions of high interest and connect contextual features from distal parts of an input. They have had a huge impact on the quality of output from deep learning networks over the last few years, and as a consequence, we decided to employ them within our Pix2PixGAN. We envisaged that their use could greatly compensate the regions of low scattering intensity. However, we were unable to find a published Pix2PixGAN featuring a transformer, and therefore in order to apply them here we ran several benchmarks to assess their applicability. We discuss the architecture of and report on the best-performing results below but note we were unable to complete a comprehensive suite of benchmarks owing to our limited computational resources.

Against the generator architecture provided in Table 2, the primary difference is the insertion of a self-attention layer after every block with the exception of Down 0 and Up 4 and 5. We follow the standard procedure for self-attention on images, where the image is treated as an input sequence with length width $\times$ height. We set the number of attention heads as equal to the output channel size divided by 8. We replace the InstanceNorm operations with SpectralNorm following Silva [5]. For the discriminator, we apply a self-attention layer after Block 1 and 3, with SpectralNorm again used throughout.

A comparison of the general assessment metrics is provided in Table 4 versus the counterpoint values obtained from DSFU-net. Figure 5 shows the predicted output from the two validation examples discussed in the main text in Figure 3.

Table 4: Fréchnet Inception Distance (FID), Kernel Inception Distance (KID), and Mean Squared Error (MSE) between the DSFU-net-generated and ground truth (GT) scattering planes for the validation (12607 total samples) and training datasets (50000 random selection of samples) compared with the self-attention version of DSFU-net. The lower the score, the better.

		Validation		Training	
		DSFU-net vs. GT	Attn. vs. GT	DSFU-net vs GT	Attn. vs. GT
$I_{\mathrm{SRO}}(\mathbf{Q})$	FID	13.1	32.3	8.35	26.4
	KID	0.003	0.018	0.002	0.009
	MSE	0.015	0.043	0.010	0.035
$I_{\mathrm{FF}}(\mathbf{Q})$	FID	36.9	113.9	33.3	103.3
	KID	0.008	0.066	0.018	0.100
	MSE	0.006	0.011	0.004	0.008

In general, the transformer scores are worse than the DSFU-net counterpart. The significantly increased FID and KID scores indicate that the self-attention network is unable to capture the probability distribution of $I_{\mathrm{SRO}}(\mathbf{Q})$ and $I_{\mathrm{FF}}(\mathbf{Q})$ as proficiently as DSFU-net. Through visual inspection, we can see in Figure 5 that the transformer network output is not quite as sharp as DSFU-net, with pixelation and blurred artefacts appearing on the output. In general, while we occasionally observe some improvement in compensating for regions of low intensity such as in the top of the $I_{\mathrm{FF}}(\mathbf{Q})$ Transformer output in Figure 5, these manifested artefacts worsen the quality of our output. The use of self-attention in our U-NET generator considerably increases the number of trainable parameters, and thus these defects may be a sign of overfitting. An increased dataset size, coupled with finer-tuning of the hyperparameters, including the placement of the self-attention layers, would likely resolve these issues and potentially improve the quality of output beyond that reported here.

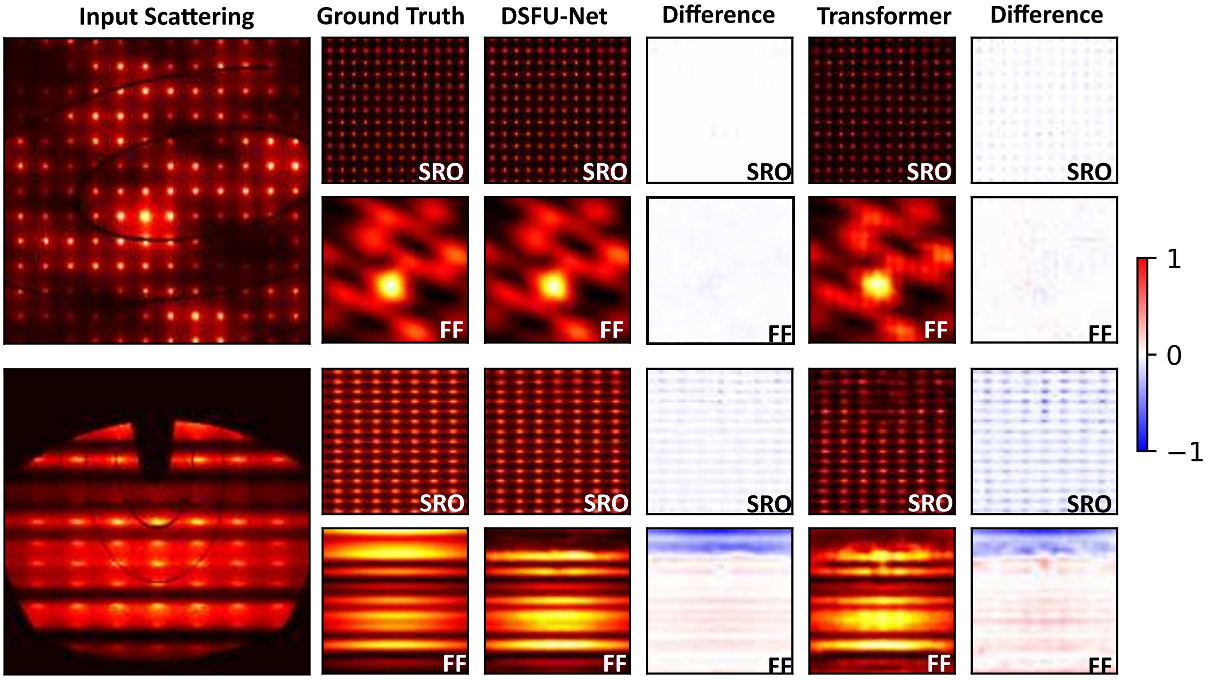

Figure 5: Two examples from Figure 3 in the main text of scattering inputs from the validation dataset and their corresponding DSFU-net/transformer GAN outputs and GTs for one of the best (top) and worst (bottom) performances. We also provide the differences between the DSFU-net and transformer GAN outputs versus the GT. Scattering planes are shown on a square root scale to better emphasise low-intensity features.

As an alternative approach to tackling the regions of low intensity, we wondered whether the decoder within the generator could be improved in terms of its ability to produce *de novo* intensity, as opposed to mapping purely from the input scattering plane, using the surrounding contextual information as a conditioning measure. We leveraged the recently published Dynamic Pix2PixGAN [6] for this task. In summary, the network maps towards the target distribution better than a traditional Pix2PixGAN owing to its dynamic neural network. It accepts as input both conditioning images (in our case the scattering planes) and pure noise. A bottleneck in the centre of the U-NET freezes the encoder when noise is input, ensuring no dependency is learnt within the encoder on noise, while the decoder can still learn the target distribution based on this noise. This bottleneck is opened when a scattering plane is used, allowing backpropagation to flow through the encoder. The discriminator is then fed either noise or the scattering plane concatenated to the $I_{\text{SRO}}(\mathbf{Q})$ or $I_{\text{FF}}(\mathbf{Q})$ as necessary. Within the context of this work, regions of very low intensity in the input scattering planes were replaced with noise to emulate the pure noise input.

The dynamic Pix2PixGAN performed worse overall than the transformer version discussed above, primarily as it would fill the noised regions with nonsensical $I_{\text{SRO}}(\mathbf{Q})$ or $I_{\text{FF}}(\mathbf{Q})$ that did not fit within the context of the GT. Consequently, we also included some self-attention layers, albeit with a considerably reduced architecture size than described above due to VRAM limitations. This network performed worse than the transformer GAN, but better than the pure dynamic Pix2PixGAN. In much of the training data, the size of the 'instrumental' artefacts varies significantly, in some cases making up most of the input scattering plane. We, therefore, attribute the worse performance to the inability of the network to correctly identify relevant contextual information. In the ideal circumstance of a training set built from clean input samplings, coupled with greater benchmarking of architectures and improved computational resources, we expect the transformer GAN to perform best. In this context, the dynamic Pix2PixGAN would be less applicable, as the addition of noise would effectively silence any contributions from small but present low-intensity regions.

2.3 Differing training strategies

We experimented with several training strategies to ensure the highest quality results from DSFU-net. We outline these briefly below but note that none ended up in the final version of DFSU-net.

1. We trained both the generators or discriminators for additional iterations (4, 5, 6 times were attempted) while training the other for only one iteration to prevent either the generator or discriminator from becoming too strong. Unsurprisingly as the losses are well-behaved, this had minimal impact on the quality of the output, only increasing the computational cost of training.

2. A layer of Gaussian noise was included in each Block of the discriminator, emulating adding a gradient penalty to the discriminator loss function. This acts to weaken the discriminator, avoiding the loss appearing as a step function and ensuring the generator has a gradient to learn from. Similar to the additional training iterations, this had little impact on the quality of the output. Note we also included this with the transformer GAN, but again found it had little impact.

3. As opposed to the loss described in Equation 8 of the main text, we applied a WGAN type loss, which effectively uses a Wasserstein distance measure (see Equation 5) coupled with a gradient penalty term [7]. This approach ensures the generator always has a vector direction to adjust its parameters towards - in other words, the loss in the main text presumes there is already some overlap between the initial and final probability distributions to use as a guide, which can be problematic, particularly at the start of training.

4. We ran an ablation test on the use of our auxiliary loss described in Equation 11, effectively to test the quality of the output from the two decoupled GANs. We found that while output was still reasonable, the network generalisability worsened, a property particularly noticeable in regions of low scattering intensity. Thus, the addition of this loss aids in the network's learning of the factorisation and ability to compensate for missing data.

3 Analysis of DSFU-net output

3.1 Performance assessment

The Fréchnet Inception Distance (FID) [8] and Kernel Inception Distance (KID) [9] are both means of assessing the quality of images produced *via* generative models as an effective proxy for human assessment. They are typically applied to compare generative models and measure performance. While no formal comparison can be made here as this work is the first of its kind, we can still use the absolute value to inform us of the perceived quality of the model's output and, more specifically, assess the model's performance on the validation dataset to examine whether it is generalised enough. The FID measures the differences in density between two distributions of assumed Gaussian shape based on the pre-trained InceptionV3 classifier [10]. Specifically, it calculates the Fréchnet distance between two multivariate Gaussians fitted to the real and generated data.

Since we don't necessarily know that our distributions are Gaussian, we also employ the KID as a comparative measure. KID measures the maximum mean discrepancy between InceptionV3 embeddings of the real and generated data using a polynomial kernel; in other words, it does not assume a Gaussian prior. The KID is generally, therefore, a more generalisable metric, but we include the FID as it is a more widely-used scoring method.

It is worth noting that since the FID and KID both use a classifier network trained on a large set of images which do not feature examples like our training data, it is unlikely that these metrics will accurately capture all underlying features present in our scattering data. However, since the training set for these classifiers is very general - the ImageNet dataset [11] - it is a safe assumption that these metrics can approximate the correct distance between our target and source domains.

We employ the pixel-wise mean-squared error (MSE) as a third metric to measure the similarity between the ground truths and the network-generated images. While it is unable to measure distances between the probability distributions DSFU-net is trained to recapitulate, it is simpler to interpret. The MSE was calculated for each sample in the validation dataset for the FF and SRO components. Because the magnitude of the error depends on the magnitude of the pixel intensities, the MSE between the ground truths and some random noise (normalised between -1 and 1) was calculated along with the MSE between two similarly-sized datasets of random noise to contextualise our values. The resulting MSE distributions for the FF and SRO are shown in Figure 6.

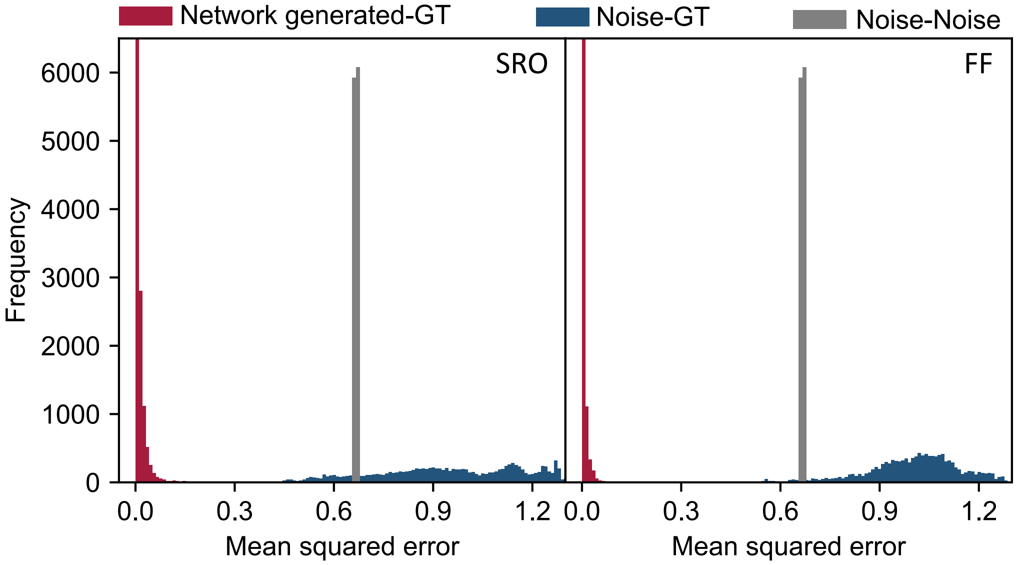

Figure 6: Histograms of the pixelwise mean squared error between various test datasets.

3.2 Tris-*tert*-butyl-1,3,5-benzene tricarboxamide

The data used in this work was originally collected by Kristiansen *et. al.* who solved the structure from single crystal data. An illustration of the average structure and the disorder is given below for reference, along with a table with fractional coordinates of the asymmetric unit cell in Table 5.

Table 5: Fractional coordinates of the asymmetric unit cell of tris-*tert*-butyl-1,3,5-benzene tricarboxamide. The lattice parameters are $a = b = 14.1212$ Å, $c = 6.9341$ Å, $\alpha = \beta = 90°$, $\gamma = 120°$ and the space group is $P6_3/m$.

Atom	x	y	z
O	0.77740	0.72910	0.01110
N	0.70500	0.80110	0.21800
C	-0.09230	0.01070	0.13190
C	0.89670	-0.09240	0.13280
C	0.58910	0.70930	0.20900
C	0.78730	0.80500	0.11400
C	0.58390	0.60620	0.28600
C	0.52580	0.74340	0.33880
C	0.54820	0.69190	0.00400
H	0.72000	0.85470	0.29510
H	0.84420	0.01900	0.12100
H	0.61020	0.61820	0.41650
H	0.62870	0.58800	0.20740
H	0.50950	0.54700	0.28280
H	0.55320	0.75200	0.46820
H	0.44970	0.68830	0.33660
H	0.53420	0.81170	0.29430
H	0.55060	0.75720	-0.04260
H	0.47420	0.63210	-0.00050
H	0.59370	0.67530	-0.07560

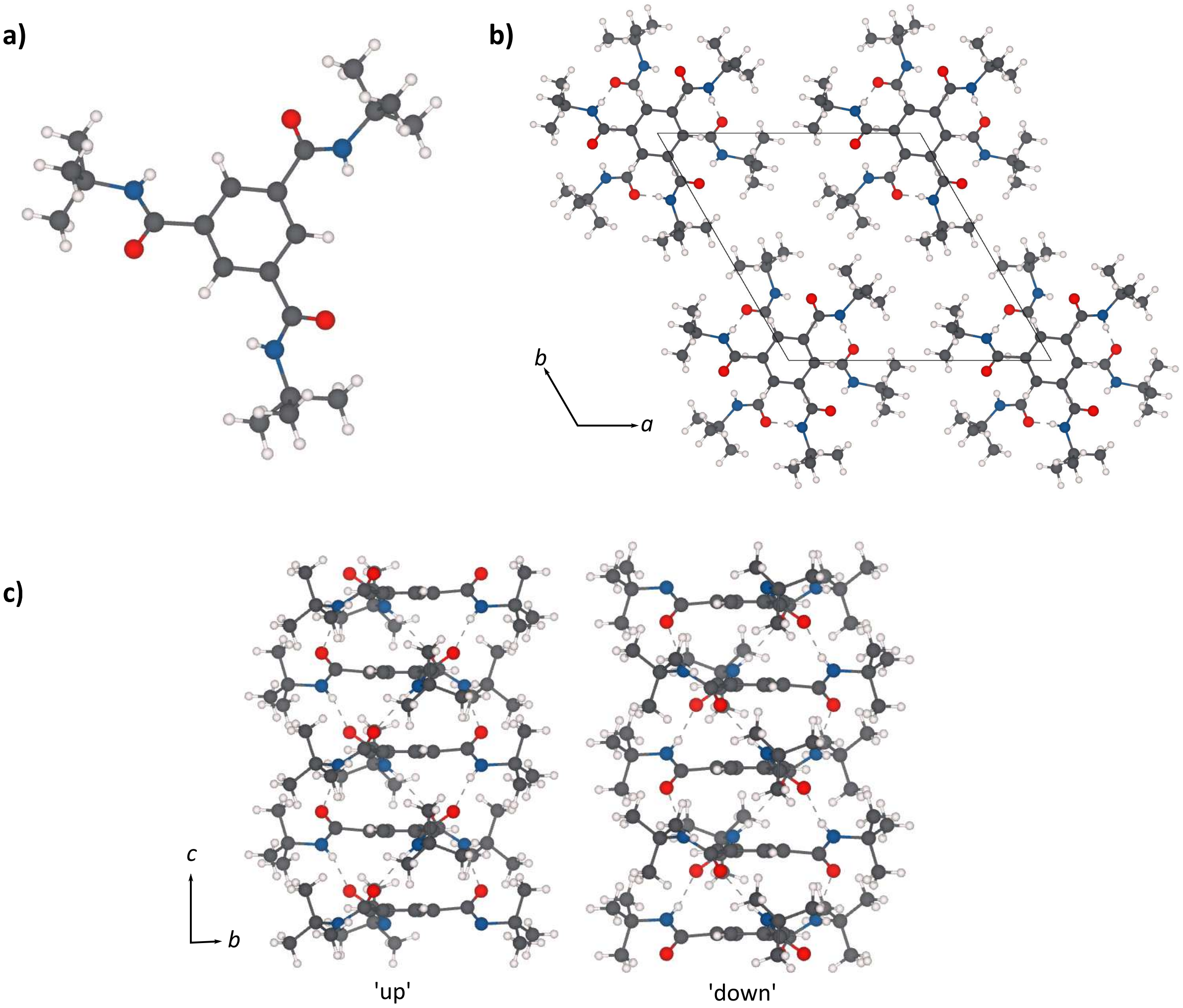

Figure 7: a) A single tris-*tert*-butyl-1,3,5-benzene tricarboxamide molecule with carbon, nitrogen, oxygen and hydrogen shown in grey, blue, red and white, respectively. b) The molecule in its unit cell, viewed down the c-axis and along the molecular stacks. c) The two possible orientations of the molecular stack, with the oxygen atoms above (up) or below (down) the plane of the central benzene ring.

This molecule has been used previously by a few members of the diffuse scattering community to test their analysis methods [12, 13]. The data used here were kindly provided by Arkadiy Simonov, in the form of pre-processed hkx planes [13]. The background had been estimated using the average intensity of the planes just above and below those of interest, and the Bragg peaks were removed using the Punch-and-fill method.

3.3 Least-squares refinement of SRO

The first step is to project the DFSU-Net SRO output into one reciprocal space unit cell. The size of the reciprocal unit cell in pixels is determined by the resolution of the original layer reconstruction and the size of the real-space unit cell. In this example, the reciprocal unit cell is 15×17 pixels; however, because we were fitting in python and thus are limited to using square pixels, we found that is was easier and better if we used a larger rectangular unit cell of 30×17 pixels, instead of the obvious hexagonal one. The intensities were summed averaged all the unit cells in the plane and then were normalised to 1. Additional processing steps such as masking low-intensity areas were applied before this averaging.

If the mask is not included, the noise in the low-intensity areas softens the features in the SRO scattering, leading to smaller SRO parameters being refined. The SRO parameters refined from the DSFU-Net output with no preprocessing are shown in Table 6, with those from YELL in the main text repeated for convenience. Crucially, the signs of all the SRO parameters are still correct.

Table 6: A comparison between SRO parameters refined by Schmidt and Neder using YELL , and the least-squares refinement in this work with no preprocessing. Note that $\mathbf{v}$ is given here in reciprocal lattice units.

$\mathbf{v}$	YELL	This work
(1,0,0)	-0.2516	-0.1617
(2,0,0)	0.0984	0.0669
(2,1,0)	0.0950	0.0453
(3,0,0)	-0.0345	-0.0186
(3,1,0)	-0.0532	-0.0233
(3,2,0)	-0.0435	-0.0200
(4,0,0)	0.0164	0.0035
(4,1,0)	0.0256	0.0097
(4,2,0)	0.0310	0.0090
(4,3,0)	0.0165	0.0074
(5,0,0)	-0.0090	-0.0006
(5,1,0)	-0.0128	-0.0016
(5,2,0)	-0.0175	-0.0055
(5,3,0)	-0.0149	-0.0039
(5,4,0)	-0.0073	-0.0014

Another thing we noted during testing was that careful treatment of any Bragg scattering is required. Since Bragg scattering is periodic and often much more intense than any diffuse scattering, if there are Bragg peaks in the input plane, the SRO output from DSFU-Net will be a mixture of any scattering from SRO and Bragg. A simple way to remove Bragg peaks is the punch and fill method, where the intensities of n pixels around each peak are set to the average intensity of the surrounding background pixels. We found that DSFU-Net is sensitive enough to interpret this circle of constant scattering as a periodic feature, which adversely affects the the refinement of SRO parameters. Therefore, when removing the Bragg peaks, it is important to replace them with realistic noise fluctuations, so that no artificial periodic features are added.

3.4 Simulated data test

During the above analysis, it was found that the intensity of the DSFU-Net output $I_{\mathrm{FF}}(\mathbf{Q})$ did not have the same Q-dependence as the calculated $I_{\mathrm{FF}}(\mathbf{Q})$ and appeared to decrease at a faster rate. It was thought that a possible cause for this could be that the network had learnt that all $I_{\mathrm{FF}}(\mathbf{Q})$s decay with the same rate, as all of the training data provided were calculated with a very similar Q_{max} and were largely small organic molecules. To test whether this was the case, the scattering of Tris-*tert*-butyl-1,3,5-benzene tricarboxamide was calculated from its constituent SRO and FF components, on the same Q-grid as the experimental data. The $hk1$ plane is shown in Figure 8. This was input into DSFU-Net and the outputs are also shown in Figure 8, along with the correct SRO and FF components used for the initial scattering calculation.

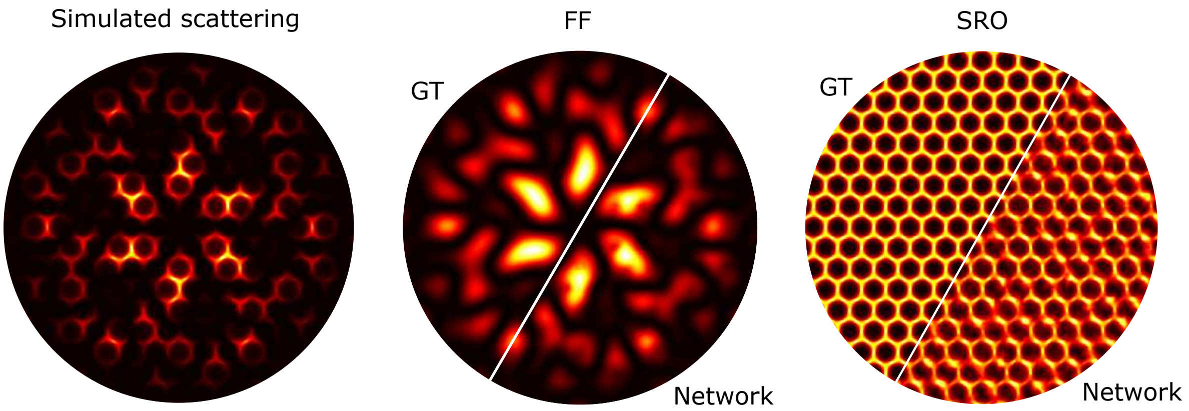

Figure 8: Simulated $hk1$ scattering plane, and the constituent ground truth (GT) FF and SRO compared with the FF and SRO predicted by the network.

In this simulated example, the network reproduces the calculated $I_{\mathrm{FF}}(\mathbf{Q})$ all the way out to $Q_{\max}$ and the intensities do not appear to drop off. This phenomena must therefore be due to the experimental data and the network has not falsely learnt a particular Q-dependence. Further discussion of this is given in the main text.

It is also worth noting that, just like with the experimental data, there are 'holes' in the intensity near the middle of the $I_{\mathrm{FF}}(\mathbf{Q})$ pattern due to the low input intensities. However, both the FF and, more noticeably, the $I_{\mathrm{SRO}}(\mathbf{Q})$ in this example look much cleaner with lower noise levels. This demonstrates the effect of experimental noise on the quality of the network output.

References

(1) Virtanen, P. et al. *Nature Methods* **2020**, *17*, 261–272.
(2) Isola, P.; Zhu, J.-Y.; Zhou, T.; Efros, A. A. Image-to-Image Translation with Conditional Adversarial Networks, 2018.
(3) Saxena, D.; Cao, J. Generative Adversarial Networks (GANs Survey): Challenges, Solutions, and Future Directions, 2023.
(4) Vaswani, A.; Shazeer, N.; Parmar, N.; Uszkoreit, J.; Jones, L.; Gomez, A. N.; Kaiser, L.; Polosukhin, I. Attention Is All You Need, 2017.
(5) Silva, T. S. *https://sthalles.github.io* **2018**.
(6) Naderi, M.; Karimi, N.; Emami, A.; Shirani, S.; Samavi, S. **2022**.
(7) Gulrajani, I.; Ahmed, F.; Arjovsky, M.; Dumoulin, V.; Courville, A. C. *CoRR* **2017**, *abs/1704.00028*.
(8) Heusel, M.; Ramsauer, H.; Unterthiner, T.; Nessler, B.; Hochreiter, S. GANs Trained by a Two Time-Scale Update Rule Converge to a Local Nash Equilibrium, 2018.
(9) Bińkowski, M.; Sutherland, D. J.; Arbel, M.; Gretton, A. Demystifying MMD GANs, 2021.
(10) Simonyan, K.; Zisserman, A. Very Deep Convolutional Networks for Large-Scale Image Recognition, 2015.
(11) Deng, J.; Dong, W.; Socher, R.; Li, L.-J.; Li, K.; Fei-Fei, L. In *2009 IEEE conference on computer vision and pattern recognition*, 2009, pp 248–255.
(12) Schmidt, E.; Neder, R. B. *Acta Crystallographica Section A* **2017**, *73*, 231–237.
(13) Simonov, A.; Weber, T.; Steurer, W. *Journal of Applied Crystallography* **2014**, *47*, 2011–2018.
